# Phenotyping of Panicle Number and Shape in Rice Breeding Materials Based on Unmanned Aerial Vehicle Imagery

**DOI:** 10.34133/plantphenomics.0265

**Published:** 2024-10-24

**Authors:** Xuqi Lu, Yutao Shen, Jiayang Xie, Xin Yang, Qingyao Shu, Song Chen, Zhihui Shen, Haiyan Cen

**Affiliations:** ^1^ College of Biosystems Engineering and Food Science, Zhejiang University, Hangzhou 310058, China.; ^2^ Key Laboratory of Spectroscopy Sensing, Ministry of Agriculture and Rural Affairs, Hangzhou 310058, China.; ^3^ The Advanced Seed Institute, Zhejiang University, Hangzhou 310058, China.; ^4^ National Key Laboratory of Rice Biology and Breeding, Zhejiang University, Hangzhou 310058, China.; ^5^ China National Rice Research Institute, Chinese Academy of Agricultural Sciences, Hangzhou 310006, China.; ^6^ Zhejiang Dahua Technology Co. Ltd., Hangzhou 310056, China.

## Abstract

The number of panicles per unit area (PNpA) is one of the key factors contributing to the grain yield of rice crops. Accurate PNpA quantification is vital for breeding high-yield rice cultivars. Previous studies were based on proximal sensing with fixed observation platforms or unmanned aerial vehicles (UAVs). The near-canopy images produced in these studies suffer from inefficiency and complex image processing pipelines that require manual image cropping and annotation. This study aims to develop an automated, high-throughput UAV imagery-based approach for field plot segmentation and panicle number quantification, along with a novel classification method for different panicle types, enhancing PNpA quantification at the plot level. RGB images of the rice canopy were efficiently captured at an altitude of 15 m, followed by image stitching and plot boundary recognition via a mask region-based convolutional neural network (Mask R-CNN). The images were then segmented into plot-scale subgraphs, which were categorized into 3 growth stages. The panicle vision transformer (Panicle-ViT), which integrates a multipath vision transformer and replaces the Mask R-CNN backbone, accurately detects panicles. Additionally, the Res2Net50 architecture classified panicle types with 4 angles of 0°, 15°, 45°, and 90°. The results confirm that the performance of Plot-Seg is comparable to that of manual segmentation. Panicle-ViT outperforms the traditional Mask R-CNN across all the datasets, with the average precision at 50% intersection over union (AP_50_) improved by 3.5% to 20.5%. The PNpA quantification for the full dataset achieved superior performance, with a coefficient of determination (*R*^2^) of 0.73 and a root mean square error (RMSE) of 28.3, and the overall panicle classification accuracy reached 94.8%. The proposed approach enhances operational efficiency and automates the process from plot cropping to PNpA prediction, which is promising for accelerating the selection of desired traits in rice breeding.

## Introduction

Rice (*Oryza sativa* L.) ranks among the top 3 staple food crops worldwide, with over half of the global population consuming rice as a staple food, and in China, more than 65% of the population relies on rice as their primary food source, underscoring the global importance of sustaining rice production [1,2]. In the face of climate change, there is an urgent need to develop new rice cultivars that are resilient to stress, high yield, and high quality [3–5].

During the rice breeding process, the panicle number per unit area (PNpA), one of the 4 key components of yield, is a trait of substantial interest to breeders. It plays a crucial role in assessing genetic gains, yield formation, and yield production [6–8]. Traditionally, in rice breeding, PNpA is determined through field sampling surveys, where an average of the total panicles from 6 selected plants per plot is calculated to estimate the PNpA for that plot on the basis of breeders’ recommendation [9]. However, this method poses issues such as sample bias and statistical significance, especially since the number of plants per plot can range from several dozen to hundreds, and the extensive number of materials makes manual counting time consuming and labor intensive [10]. While recording PNpA, breeders also simultaneously document the curvature characteristics of the panicle type, which are typically classified into erect and curved panicles in general.

Although studies on small-scale panicle detection via indoor or fixed observation platforms and computer vision have been conducted, the efficiency of image collection is low, making it difficult to apply in large-scale field breeding trials [11]. More importantly, throughout the growth process of many different rice breeding materials, significant changes in the shape, color, size, texture, and posture of panicles occur, making large-scale field phenotypic analysis of rice panicles challenging at the right time [1,12,13]. Additionally, considering the practical aspects of breeding, breeders often plant hundreds to thousands of breeding materials across multiple locations at the same time to assess crop geographical adaptability and accelerate the breeding process, necessitating a high-throughput method for data collection and efficient and accurate automatic data analysis for rapid and precise evaluation of the required parameters within a short time window [14].

In terms of high-throughput acquisition of field rice canopy images, methods include satellite remote sensing, light aircraft, and unmanned aerial vehicles (UAVs) equipped with imaging devices [15]. Each of these data acquisition methods has advantages and disadvantages. For example, satellite remote sensing operates on a large scale but struggles to collect small target objects such as rice panicles and is subject to interference from clouds, resulting in variable data collection frequency and quality [16,17]. Light aircraft, typically equipped with hyperspectral and multispectral image sensors, operate at high altitudes and speeds, which complicates the collection of clear images of panicle targets [18]. On the other hand, low-altitude remote sensing with UAVs carrying high-resolution RGB cameras offers new opportunities for panicle phenotyping because of their good flexibility, high spatial resolution, and relatively low cost [19,20]. Currently, there are 2 main approaches for acquiring RGB images through low-altitude remote sensing with UAVs. The first involves UAVs capturing images at a fixed point near the canopy, directly obtaining plot-scale images [21–23]. The drawback of this method is its low imaging efficiency, and the wind generated by UAV operations could adversely affect rice growth, making it less practical for breeding applications. The alternative approach balances efficiency and image quality by selecting an appropriate near-ground height for UAVs to execute flight missions efficiently, followed by segmenting large images into plots [15,24], which makes high-throughput phenotyping of rice panicles in breeding programs possible.

With high-quality image data, object detection and instance segmentation methods based on deep learning are also necessary for the panicle detection task. Owing to the ability of deep learning to leverage multiple hidden layers to extract advanced features, it has demonstrated superiority in reliably classifying, labeling, and segmenting targets, particularly in panicle segmentation tasks [25]. Xiong et al. [26] developed a superpixel segmentation and convolutional neural network (CNN)-based model, Panicle-SEG-CNN, which effectively segments rice panicles. Xu et al. [27] introduced the multi-scale hybrid window panicle detect (MHW-PD) model utilizing Faster R-CNN, and Hong et al. [28] proposed the Panicle-Mask algorithm based on Mask R-CNN for detailed panicle region quantification. Sun et al. [29] improved YOLOv4 for curved panicle detection, whereas Tan et al. [13] introduced RiceRes2Net, which is based on an enhanced Cascade R-CNN, for field detection and growth stage identification of rice panicles. These methods excel in extracting local features such as edges and textures but may fall short in capturing the intricate interplay of low-level (e.g., morphology and color) and high-level (e.g., semantic information of objects and scenes) features, whereas employing the vision transformer (ViT) as the backbone network may offer significant advantages [30–32]. Originating from natural language processing tasks, ViT boasts superior global perception capabilities, scalability, and generalization abilities compared with traditional CNNs [33–35]. Since the mask region-based CNN (Mask R-CNN) is widely used in instance segmentation tasks and is capable of precise counting, it serves as a fundamental model for our panicle detection task.

In summary, this study aims to develop a high-throughput, plot-level rice PNpA quantification method for field breeding based on low-altitude remote sensing with UAVs. The specific objectives are as follows: (a) to acquire rice field canopy images at suitable altitudes via flight missions and establish a method for automatic field plot segmentation; (b) to combine ViT and Mask R-CNN to construct a Panicle-ViT instance segmentation network for accurate PNpA parameter evaluation of individual plot images; and (c) to develop a new set of classification standards and classifiers for panicle curvature, providing breeders with more comprehensive phenotypic information on rice panicles.

## Materials and Methods

### Experimental design

**Fig. 1.**
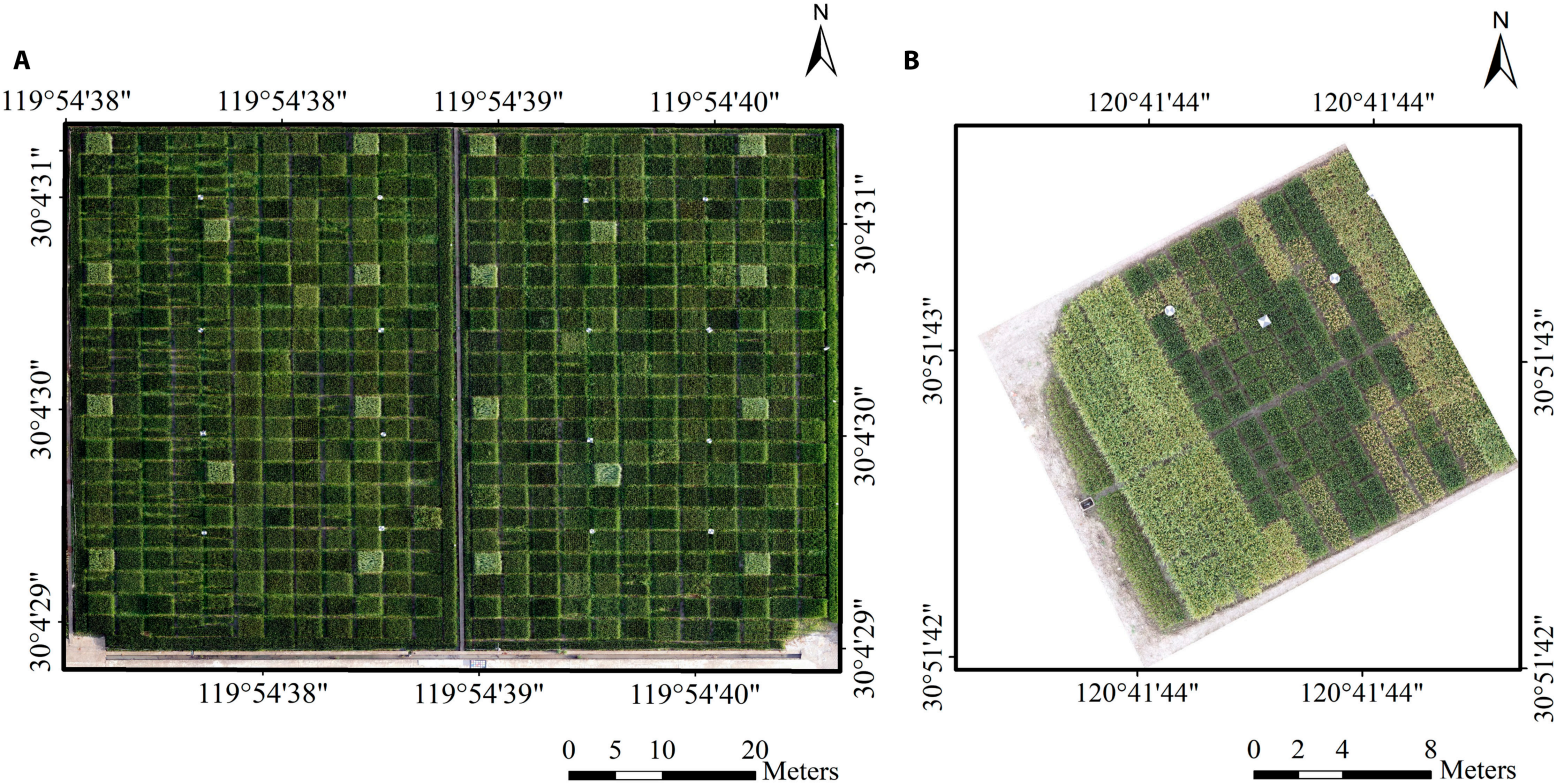
Study sites located at the China National Rice Research Institute, Hangzhou, Zhejiang Province, China, 98 d after rice sowing (A) and at the Jiaxing Academy of Agricultural Sciences, Jiaxing, Zhejiang Province, China, 104 d after rice sowing (B).

In this study, a rice breeding trial was conducted in 2022 at the experimental field of the China National Rice Research Institute (30°4′29.5″N, 119°54′37.3″E), which is located in Hangzhou, Zhejiang Province, China (field 1), as shown in Fig. 1. The experiment comprised 550 plots, including 470 planted with double-haploid (DH) line materials derived from doubled haploid breeding (DHB). Furthermore, 4 standard rice varieties, including Tianyouhuazhan, Zhejing 99, Jiangliangyou 7901, and Yongyou 1540, were cultivated, with each variety replicated across 20 plots, resulting in a total of 80 plots. These 4 standard varieties were specifically used as controls to demonstrate the potential variations in fertilization and watering conditions across the breeding field. These DH lines, characterized by their stable traits and ability to produce pure lines through selfing, can be consistently propagated and utilized over extended periods [36,37]. Each plot with an area of 2.4 m × 2 m was arranged with a spacing of 20 cm × 20 cm and an interplot spacing of 40 cm × 40 cm. Rice sowing took place in mid-May, transplanting in mid-June, and harvesting in October. Fertilization followed standard management practices, including 120 kg/ha of nitrogen, 60 kg/ha of phosphorus, and 90 kg/ha of potassium fertilizers.

To investigate the robustness of the model in detecting panicles across different years and locations of rice breeding materials, we conducted an independent experiment in the rice breeding experimental fields of the Jiaxing Academy of Agricultural Sciences (30°51′42.6″N, 120°41′44.2″E) in Zhejiang Province in 2023 (field 2). The rice breeding materials used in this experiment were obtained through hybrid breeding, differing from the DH lines developed in 2022, thus providing a better validation of the model. The plots were planted with an area of approximately 1 m × 1 m, with a plant spacing of 13 cm × 13 cm and an interplot spacing of 30 cm × 30 cm.

### Data collection

#### UAV-based data collection

The DJI M300 RTK, provided by Shenzhen DJI Innovation Technology Co. Ltd., was employed for the UAV imaging campaigns. The UAV has a high-precision RTK module, which plays a crucial role in ensuring the accuracy needed for subsequent image mosaicking processes. Additionally, the DJI Zenmuse RGB P1 camera with a resolution of 8,192 × 5,460 pixels and equipped with a 35.9 mm × 24 mm full-frame sensor alongside a DJI DL 35 mm lens was utilized and achieved a ground resolution of 1.9 mm/pixel from a 15-m flight altitude.

During the 2022 rice growth stage in field 1, UAV images were acquired at 98, 104, 111, and 125 d after rice sowing, and each campaign was conducted between 10:00 AM and 2:00 PM local time on clear, cloudless days. To ensure thorough coverage and optimal image quality, the UAV flew at an altitude of 15 m with a flight speed of 1.8 m/s while maintaining lateral and forward overlaps of 80% and 70%, respectively. In 2023, orthoimages of 100 plots were collected at field 2 on day 104 after sowing in 2023, with parameter settings consistent with those of 2022.

Additionally, to examine the impact of camera resolution on the accuracy of panicle detection, we employed an ultrahigh-resolution RGB camera (iXM-100, Phase One, Copenhagen, Denmark) with a resolution of 11,664 × 8,750 pixels, which was equipped with an 80-mm AF lens (RSM, Phase One, Copenhagen, Denmark), to capture ultrahigh-resolution orthophotos at field 1 on day 98 after sowing in 2022. The ground resolution of these ultrahigh-resolution images reached 0.71 mm/pixel by flying at an altitude of 15 m.

#### Plot manual sampling for PNpA

In traditional rice breeding, the PNpA parameter is typically calculated by selecting 6 representative rice plants within a plot, counting the total number of panicles, and then dividing by the area. To validate the advantages of our method over the traditional manual sampling approach, we selected 6 rice plants twice from plots with 120 plants across 222 cultivars to calculate the PNpA at field 1 in 2022. The stability of the samples was assessed via the mean, standard deviation, Wilcoxon test, *P* value, and correlation of the PNpA values from the 2 samples [38]. The Wilcoxon test assumes that both samples are from the same population and aims to test whether 2 independent samples are drawn from populations with the same distribution. A *P* value less than 0.05 is considered statistically significant, indicating a significant difference between the 2 samples; i.e., the samples do not have the same distribution.

### Plot segmentation via the Mask R-CNN

**Fig. 2.**
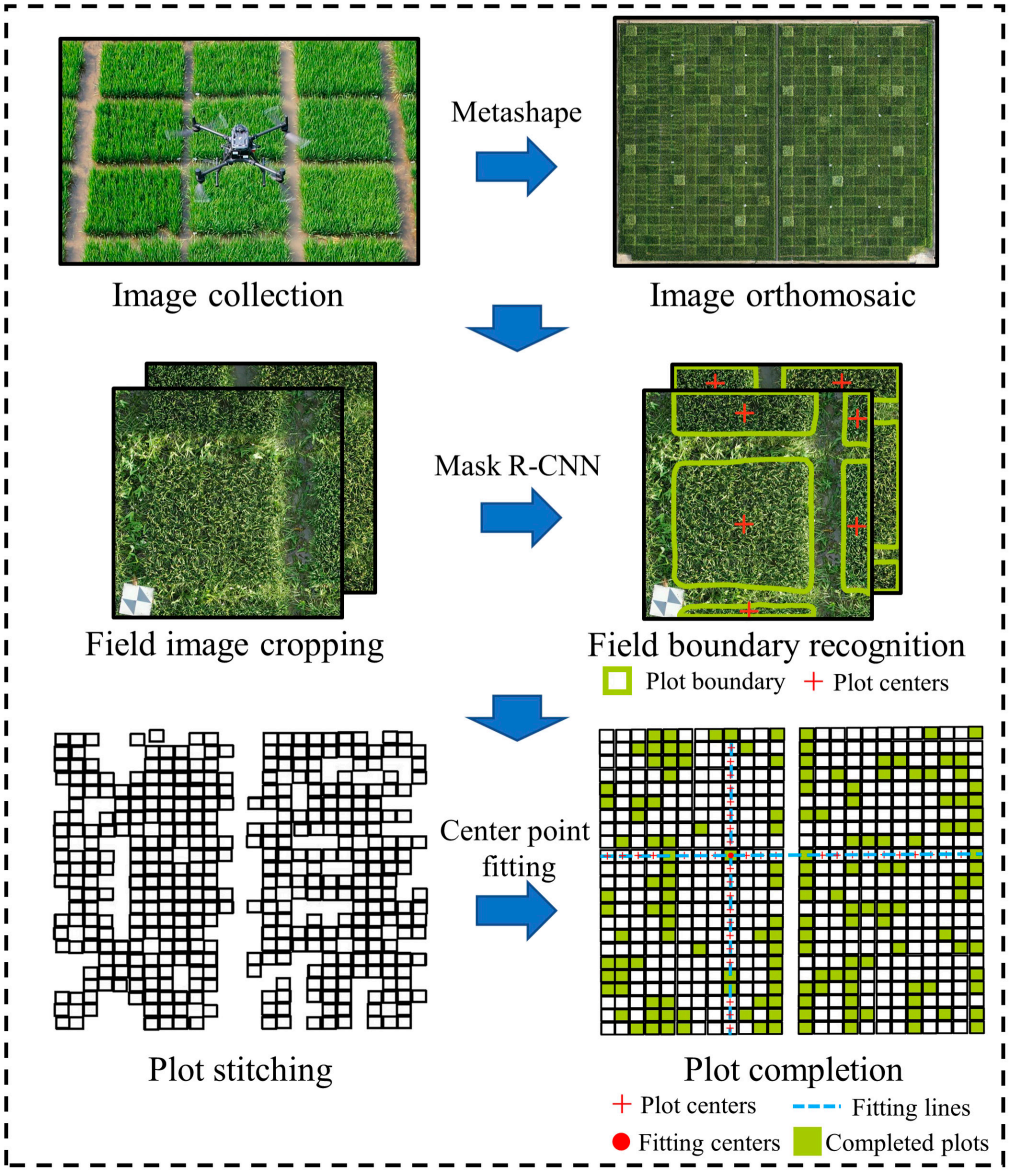
Plot segmentation in agricultural remote sensing images via the Mask R-CNN.

We developed an automated approach, named Plot-Seg, for the segmentation of experimental plots in rice fields. This method leverages the Mask R-CNN framework to identify plot boundaries accurately in UAV-captured images. The specific process is illustrated in Fig. S1 and Fig. 2. UAV images acquired from the rice field were initially processed via Metashape (Agisoft Metashape 1.7.0, St. Petersburg, Russia) to create a mosaicked image of the whole rice field. MATLAB (MATLAB 2021a Inc., Natick, MA, USA) was then used to rotate the large-field image into horizontal and vertical orientations, facilitating subsequent linear fitting and completion. The large image was subsequently cropped into smaller sections of 1,600 × 1,600 pixels, with a ^1^/_4_ overlap between adjacent sections. Mask R-CNN was applied to detect plot boundaries within these smaller images. The smaller images with detected plot boundaries were subsequently reassembled, maintaining a ^1^/_4_ overlap. When the overlap between 2 small images in a plot exceeded 60%, the images were merged, assuming that they represented parts of the same plot. This process resulted in a comprehensive rice field image annotated with plot boundaries. Finally, the central points of the detected plot areas were used to fit the horizontal and vertical lines, with their intersections determining the centers of any plots that were initially missed. The final boundaries of each plot were determined via these center coordinates and the average size of all the detected plots.

To demonstrate the precision of Plot-Seg in detecting plot boundaries, this study juxtaposed manually annotated plot boundaries with those detected by the proposed algorithm. Three annotators independently marked the boundaries of the same 10 plots. The mean centroid coordinates from the annotations of the same plot were used as the ground truth centroid coordinates, and the mean boundary point coordinates were considered the ground truth boundary point coordinates. The centroid accuracy was determined by the Euclidean distance between the centroids annotated by the algorithm, and those annotated by each annotator were compared with the ground truth. The boundary box precision was ascertained by the intersection over union (IoU) between the boundary boxes annotated by the algorithm and those by each annotator against the ground truth [39,40]. Furthermore, the standard deviation of the centroid errors across the 10 plots and the standard deviation of the boundary box IoU were computed via Eqs. 1 and 2 as follows:

$$d\left(P,Q\right)=\sqrt{{\left(x_{2}-x_{1}\right)}^{2}+{\left(y_{2}-y_{1}\right)}^{2}}$$ (1)

$$\mathrm{IoU}=\frac{A\cap B}{A\cup B}\times 100\%$$ (2)

where *P* and *Q* represent points within a 2-dimensional space, with respective coordinates (*x*_1_, *y*_1_) and (*x*_2_, *y*_2_), and where *d*(*P*, *Q*) signifies the Euclidean distance between these 2 points. *A* and *B* correspond to the ground truth bounding box and the predicted bounding box, respectively.

### Dataset preparation

#### Data categorization by time period

**Fig. 3.**
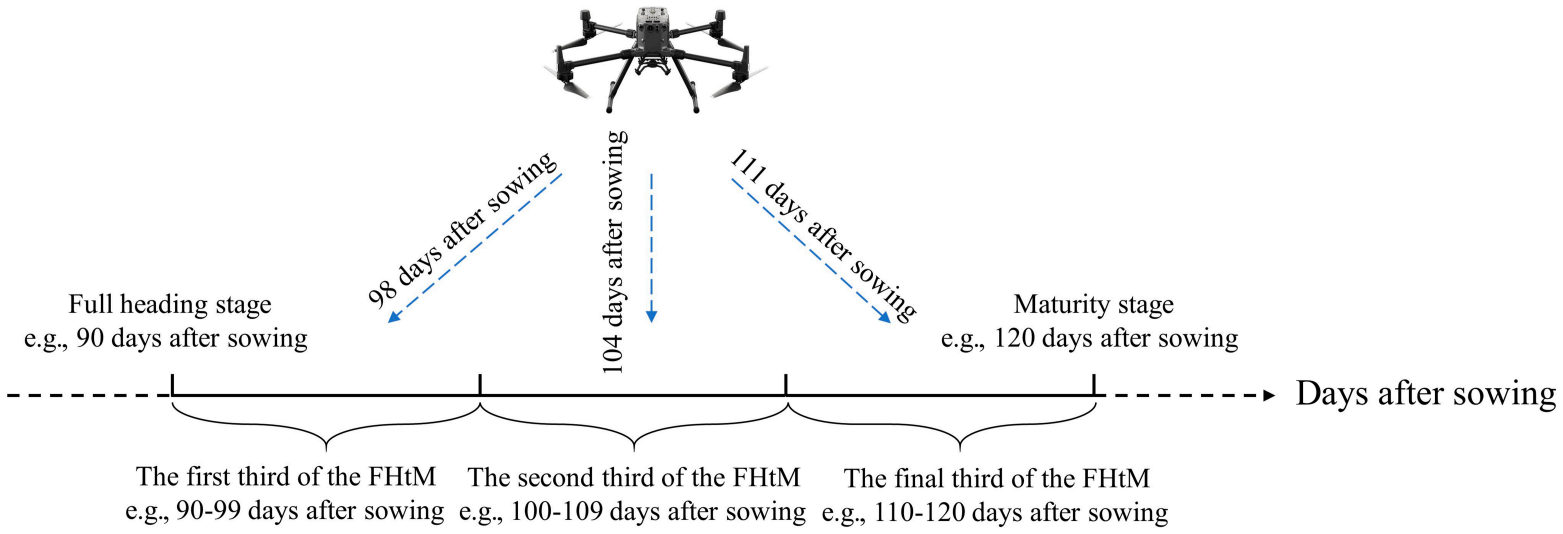
Panicle dataset categorization diagram, where FHtM represents the full heading to the maturity phase.

**Fig. 4.**
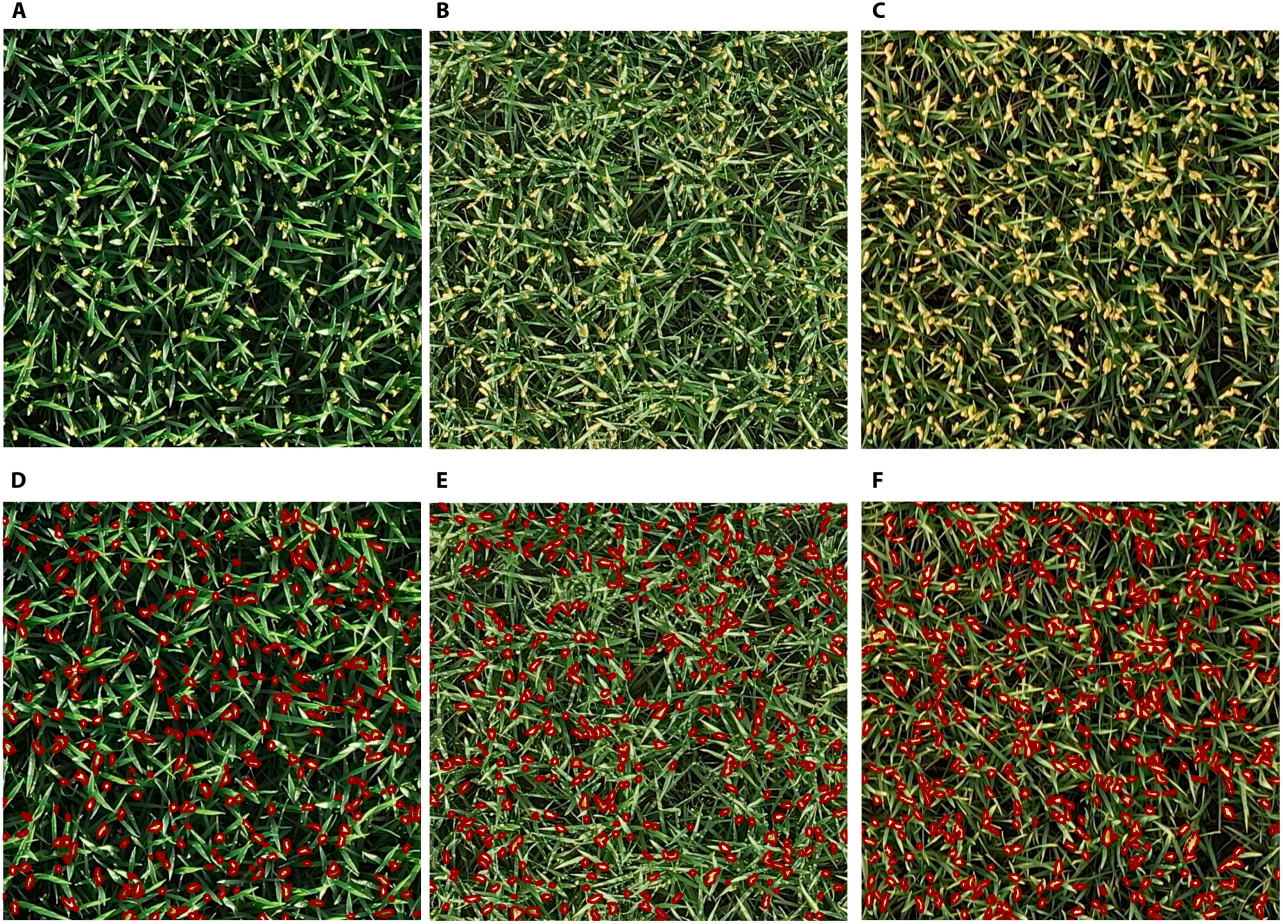
Dataset images at different stages of the FHtM. (A) Original image and (D) annotated image at the first third stage of the FHtM. (B) Original image and (E) annotated image at the second third stage. (C) Original image and (F) annotated image at the final third stage.

In the 2022 experiment in field 1, UAV data collection occurred 4 times between August and October. Given the considerable variation in growth stages among various breeding materials, relying on specific collection dates for image sampling and annotation has proven to be ineffective. Moreover, such an approach hindered the evaluation of PNpA detection performance across different growth stages. Consequently, this study conducted a statistical analysis of the heading and ripening times of 550 rice varieties. A panel of breeders recorded the rice full heading stage (FHS) and maturity stage (MS) in 550 experimental plots from August to October. The FHS is defined as the point at which the rice heading ratio reaches 80% within the plot [41]. MS is characterized by well-filled rice grains and extensive yellowing of the leaves [42]. The plant materials were then divided into 3 stages on the basis of the average date from full heading to maturity, including the first third, second third, and final third of the full heading to maturity phase (FHtM). For example, as shown in Fig. 3, if a specific rice material headed 90 d after sowing and matured after 120 d, resulting in an FHtM period of 30 d, then the UAV images collected on day 98 after sowing would fall into the first third of the FHtM. The images collected on day 104 were classified into the second third, and those collected on day 111 were classified into the final third. Images collected on day 125 after sowing were not included in the dataset. This approach was adopted to explore the detection efficacy of PNpA at different stages. Representative images of the same rice plot at 3 different stages of the FHtM are shown in Fig. 4.

#### Dataset annotation

On the basis of the segmentation results of Plot-Seg and the 3 stages delineated from heading to maturity, a total of 718 images were obtained for the first third of the FHtM, 470 images for the second third, and 249 images for the final third. Approximately 20% of the images from each stage were selected for annotation via LabelMe 5.0.1 (https://github.com/labelmeai/labelme) for panicle instance segmentation. Ultimately, 294 images of 700 × 700 pixels were annotated, with 138 images from the first third of the FHtM, 96 images from the second third, and 60 images from the final third. These datasets were divided into a training set, a validation set, and a testing set at a ratio of 6:2:2. Details of the dataset are presented in Table S1. To illustrate that the image count does not skew the results between stages, we randomized the selection of 60 images from both the first- and second-third-stage datasets, matching the quantity in the final third-stage dataset. The comparative results are displayed in Fig. S2. Figure 4D to F depicts the annotated results of rice panicles from the same rice plot at the 3 distinct stages of the FHtM. Additionally, the images from the 100 plots in 2023 were annotated to obtain 100 images as the 2023-Val dataset for the validation of model robustness. These datasets were divided into training, validation, and testing sets at a ratio of 6:2:2. Given the smaller size of the plots in 2023 (approximately 1 m × 1.2 m), which precluded the use of 700 × 700 pixel images for training, we instead cropped 400 × 400 pixel images from the center of each plot image to facilitate the quantification of PNpA. Furthermore, we randomly selected ultrahigh-resolution images from 50 plots to examine the impact of camera resolution on model accuracy. Considering the cropping size and computational processing capabilities, the ultrahigh-resolution data were cropped to a size of 1,024 × 1,024 pixels and divided into training, validation, and testing sets at a ratio of 6:2:2.

### Panicle detection via Panicle-ViT

**Fig. 5.**
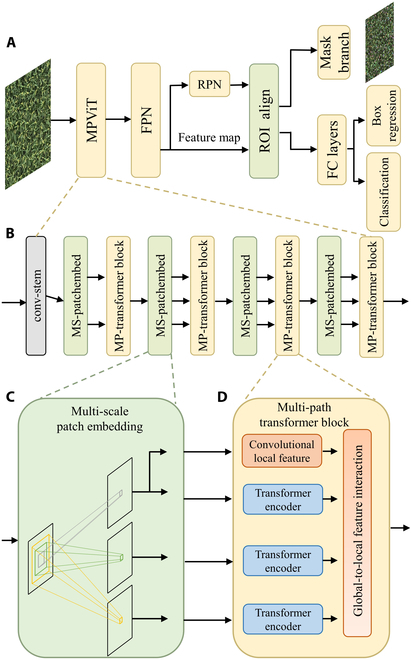
The architecture of the Panicle-ViT (A), the architecture of the backbone (B), multiscale patch embedding (C), and multipath transformer block (D).

The traditional Mask R-CNN employs a deep residual network (ResNet) as its backbone, which is notable for its ability to extract multiscale features from images [43]. However, its reliance on convolutional and pooling layers for feature extraction restricts information exchange to within the receptive field, potentially limiting the acquisition of global information [44]. Furthermore, in deep networks, the successive stacking of convolution and pooling operations may gradually lead to the loss of precise positional information from the input images [45]. This can be a drawback in tasks such as dense panicle detection, where accuracy might still need enhancement. Therefore, we introduced the adoption of MPViT (multipath ViT) as the backbone network for Mask R-CNN, substituting ResNet101, to develop Panicle-ViT, a system tailored for the detection of dense rice panicles, as shown in Fig. 5A and B [46]. This system featured a multistage architecture inspired by ViT and XCiT [47–50]. Initially, it incorporated ViT, which uses a self-attention mechanism to create global connections across the entire image, facilitating the capture of global information [51]. The integration of positional encoding enhanced the preservation of location details during feature extraction, reducing the likelihood of misidentification and omissions in densely populated object images. Additionally, the architecture was inspired by CNN models for multiscale feature extraction and fusion, utilizing multi-scale patch embeddings and independent multipath transformer encoding. As shown in Fig. 5C and D, this strategy enables the attainment of both detailed and broad feature representations within the same hierarchical level, thereby enhancing the depth and breadth of feature extraction.

In addition, the performance of Panicle-ViT was compared with that of CNN for detecting densely packed panicle detection in UAV images. First, the detection capabilities of these 2 methods were assessed via average precision (AP) and average recall (AR). The AP was determined by calculating the area under the precision–recall (PR) curve, whereas the AR was computed as the mean recall across various IoU thresholds. AP and AR are commonly used precision evaluation metrics in the fields of object detection and instance segmentation. Among them, AP_50_, AP_75_, AR_50_, and AR_75_ are widely used [52–54]. AP_50_ and AP_75_ refer to the average precision at IoU thresholds of 0.50 and 0.75, respectively. These metrics evaluate the precision of object detection models by calculating the average proportion of correct predictions (true positives) out of the total number of predicted objects, considering only predictions that meet the specified IoU thresholds. Higher AP values indicate better model precision at the respective IoU levels. AR_50_ and AR_75_ denote the average recall at IoU thresholds of 0.50 and 0.75, respectively. These measures assess the model’s ability to detect all relevant instances in the dataset, considering detections valid only if they surpass the specified IoU thresholds. Higher AR values reflect a model’s greater ability to detect all actual instances without missing many at the given IoU thresholds. The metrics are defined as follows:

$$\text{Precision}=\frac{\text{TP}}{\text{TP}+\text{FP}}$$ (3)

$$\text{Recall}=\frac{\text{TP}}{\text{TP}+\text{FN}}$$ (4)

$$\mathrm{AP}=\int_{0}^{1}p\left(r\right)\mathrm{dr}$$ (5)

$$\text{AR}=\frac{1}{N}\sum_{i=1}^{N}\text{Recal}l_{i}$$ (6)

where *TP* represents the true positive, *FP* denotes the false positive, and *FN* is the false negative. *p*(*r*) represents the precision at recall rate *r*, *Recall_i_* is the recall at the *i*th threshold, and *N* is the total number of thresholds.

The accuracy was assessed by comparing the number of panicles identified with the manually annotated count via metrics such as the coefficient of determination (*R*^2^), root mean square error (RMSE), and relative RMSE (rRMSE) to evaluate the model performance in quantifying the PNpA. The specific calculation formulas are shown in Eqs. 7 to 9. Here, *n* represents the number of samples, and *y_i_*, ${\hat{y}}_{i}$, and $\bar{y_{i}}$ denote the actual measured, predicted, and average actual measured values of the parameter, respectively [55,56].

$$R^{2}=1-\frac{\sum_{1}^{n}{\left(y_{i}-{\hat{y}}_{i}\right)}^{2}}{\sum_{1}^{n}{\left(y_{i}-\bar{y_{i}}\right)}^{2}}$$ (7)

$$\text{RMSE}=\sqrt{\frac{1}{n}\sum_{1}^{n}{\left(y_{i}-{\hat{y}}_{i}\right)}^{2}}$$ (8)

$$\text{rRMSE}=\frac{\sqrt{\frac{1}{n}\sum_{1}^{n}{\left(y_{i}-{\hat{y}}_{i}\right)}^{2}}}{\bar{y_{i}}}$$ (9)

### Classification of panicle types

**Fig. 6.**
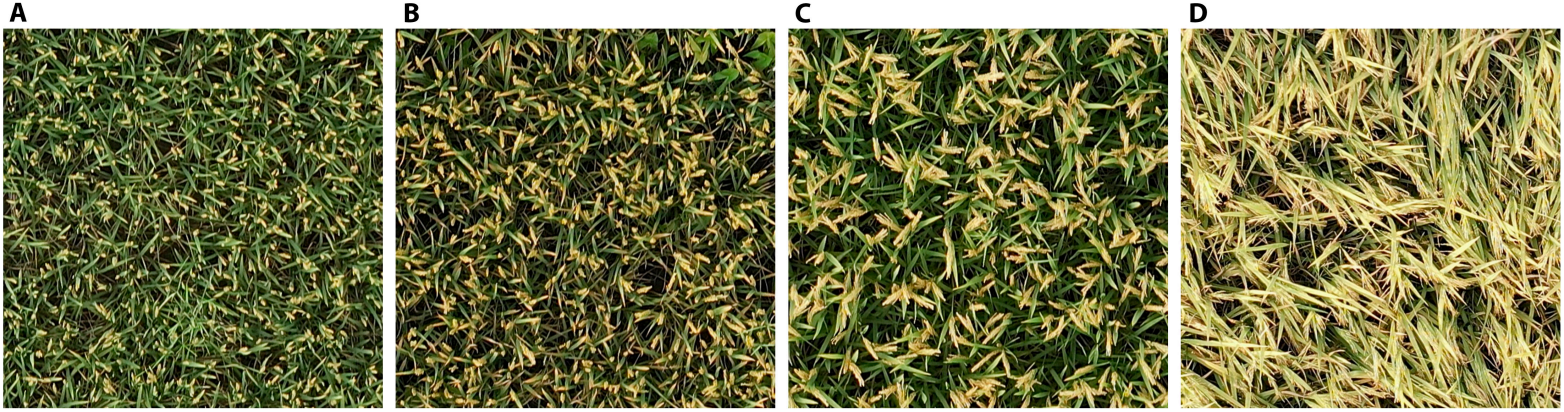
Panicles with 0° angle (A), 15° angle (B), 45° angle (C), and 90° angle (D).

In the classification of rice panicle types by breeders, panicles are generally categorized into 3 types on the basis of their curvature: erect, semi-erect, and curved [57], which is a rather coarse classification. After observing hundreds of breeding materials and considering advice from breeders, we categorized the rice panicle types into 4 categories on the basis of the angle formed between the panicle and the vertical line from the ground: 0°, 15°, 45°, and 90°, as shown in Fig. 6. The classification of rice panicles was based on images from oblique UAV photography with a camera angle of 45° relative to the ground. These images help to discern a rough range of angles at which panicles orient relative to the ground, thus categorizing them into 0°, 15°, 45°, and 90° categories, as shown in Fig. S3. This represents the first attempt to quantitatively evaluate the shape of rice panicles via UAV imagery, providing breeders with more detailed phenotypic information on rice panicles.

On the basis of the rice canopy images obtained from Plot-Seg, 719 images from the second third stage and final third stage of the FHtM (images from the first third stage were excluded because the panicle angle was not fully determined) were manually categorized into 4 types. Owing to the variation in the number of images across different panicle types, data augmentation was performed on categories with fewer images, resulting in a total of 1,917 images. This included 419 images with a 0° angle, 498 images with a 15° angle, 530 images with a 45° angle, and 470 images with a 90° angle. The dataset was then divided into training, validation, and testing sets at a ratio of 6:2:2, and the Res2Net50 classifier was employed for training and prediction [58]. Res2Net represents an evolution of the ResNet architecture, introducing the concept of multiscale feature response by transforming single-scale feature representations into multiscale feature representations. This approach captures richer image details and feature information, significantly enhancing performance in image classification tasks. The model performance was evaluated by the accuracy, precision, recall, and F1 score on the testing dataset, and the equation is as follows [59,60]:

$$\text{Accuracy}=\frac{\text{TP}+\text{TN}}{\text{TP}+\text{TN}+\text{FP}+\text{FN}}$$ (10)

$$F1\;\text{score}=2\times \frac{\text{precision}\times \text{recall}}{\text{precision}+\text{recall}}$$ (11)

where TN and FN represent true negatives and false negatives, respectively.

## Results

### Performance of the Plot-Seg

On the basis of the average centroid and boundary coordinates of 10 plots annotated by 3 manual annotators, considered the ground truth, the plot segmentation results of annotators A, B, and C and the algorithm are presented in Table 1. The mean Euclidean distance errors between the centroids of the plots annotated by A, B, and C and the ground truth are 7.71, 12.90, and 13.93 pixels, respectively. The segmentation error of the algorithm averages 28.23 pixels, which is slightly greater than that of manual annotations but still within the same order of magnitude. The average IoU values for manually annotated plots compared with the ground truth are 96.67%, 94.32%, and 92.13%, respectively, with the algorithm’s average IoU at 90.54%, which is slightly lower than the manual results but exceeds 90%.

**Table 1.** Euclidean distance, IoU, and standard deviation (SD) for algorithmic segmentation and manual segmentation. The Euclidean distance denotes the average distance between the centroids of 3 manually labeled plots, considered the true value, and the centroids derived from both manual and algorithmic labeling. The IoU represents the intersection over union between the centroids and boundaries of the 3 manually labeled plots, which serve as the true value, and the corresponding centroids and boundaries from manual and algorithmic labeling. A, B, and C represent 3 distinct individual comparisons

Methods		Euclidean distance (pixel)	SD of distance (pixel)	IoU (%)	SD of IoU (%)
Manual segmentation	A	7.71	4.41	96.67	0.98
	B	12.90	12.49	94.32	2.87
	C	13.93	14.74	92.13	3.62
Plot-Seg		28.23	13.45	90.54	2.34

Examining the standard deviation of the Euclidean distance errors for the centroids, the plot segmentation error aligns with manual annotations, with values of 13.45 pixels for the algorithm and 4.41, 12.49, and 14.74 pixels for manual annotations. Similar conclusions are drawn from the IoU with the ground truth, where the plot IoU variability of the algorithm is 2.34%, and the manual annotations are 0.98%, 2.87%, and 3.62%. As the ground truth is based on the average of 3 manual annotators, it is expected that manual annotations have smaller centroid errors and higher IoUs, and Plot-Seg achieves a result comparable to that of manual annotation. The standard deviation of the results also indicates that the proposed Plot-Seg algorithm exhibits relatively small variation compared with that of manual annotations.

**Fig. 7.**
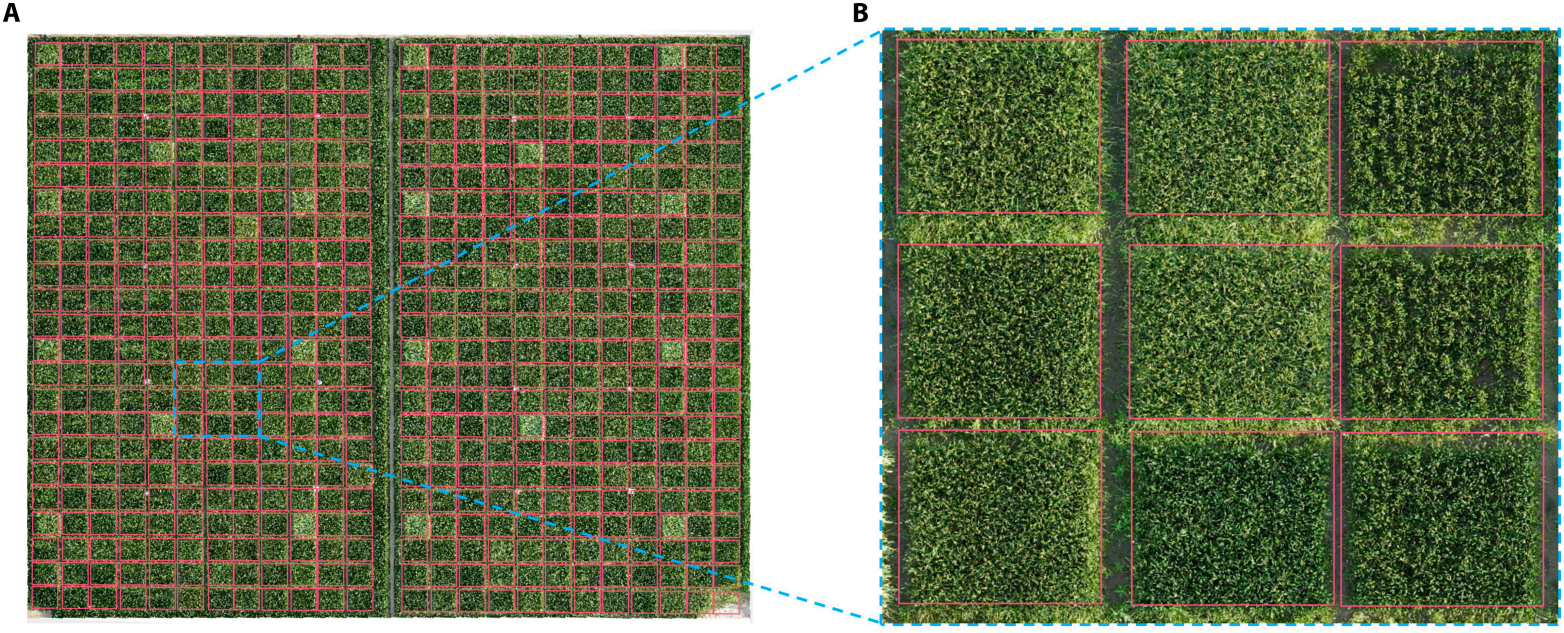
Overall results of Plot-Seg in field 1 in 2022 (A) and details of partial plot segmentation (B).

The overall segmentation results of Plot-Seg in field 1 for the year 2022 are shown in Fig. 7A, where the algorithm accurately identified each plot. Details of the segmentation for select plots are highlighted in Fig. 7B. The segmentation results for 2023 field 2 by Plot-Seg are displayed in Fig. S4.

### Comparison between Panicle-ViT and Mask R-CNN for panicle detection

To thoroughly investigate the advantages of Panicle-ViT over the Mask R-CNN, a comparison was conducted in terms of 2 main aspects: the accuracy of identification and the quantification of PNpA. Initially, the accuracy of panicle detection was compared across both the all-stage dataset and the segmented datasets by stage.

As shown in Table 2, compared with manually annotated panicle labels, the Panicle-ViT model outperformed the Mask R-CNN across all metrics in images from both the entire dataset and each stage of the FHtM. The increase in AP_50_ ranged from 3.5% to 20.5%, AP_75_ improved from 34.0% to 75.2%, AR_50_ increased from 2.2% to 15.7%, and AR_75_ increased from 7.9% to 42.1%. These results demonstrate the significant advantage of Panicle-ViT in terms of panicle detection accuracy. When Mask R-CNN was used, the worst detection accuracy occurred during the second third stage of the FHtM, with an AP_50_ of 0.541. The detection accuracies in the first third stage and final third stage of the FHtM were generally similar, with the final third stage slightly outperforming the second third stage in terms of AP_50_ and AR_75_, whereas AP_75_ was slightly lower than that in the second third stage. In the case of Panicle-ViT, the detection accuracies for all-stage images and the final third stage of the FHtM were almost identical, with AP_50_ values exceeding 0.66, surpassing those of the first third and second third stages. The visualization of Panicle-ViT for the 4 types of panicles is shown in Fig. S5.

**Table 2.** Evaluation of rice panicle detection accuracy for Mask R-CNN and Panicle-ViT. AP_50_/AP_75_ represents the average precision when the IoU is above 50%/75%, whereas AR_50_/AR_75_ represents the average recall when the IoU is above 50%/75%.

Image dataset	Network	AP_50_	AP_75_	AR_50_	AR_75_
All-stage	Mask R-CNN	0.600	0.152	0.660	0.273
The first third stage		0.614	0.186	0.683	0.302
The second third stage		0.541	0.153	0.631	0.282
The final third stage		0.616	0.161	0.683	0.303
All-stage	Panicle-ViT	0.661	0.242	0.738	0.388
The first third stage		0.635	0.266	0.698	0.385
The second third stage		0.652	0.205	0.730	0.344
The final third stage		0.662	0.282	0.726	0.327

### Performance on panicle counting

**Fig. 8.**
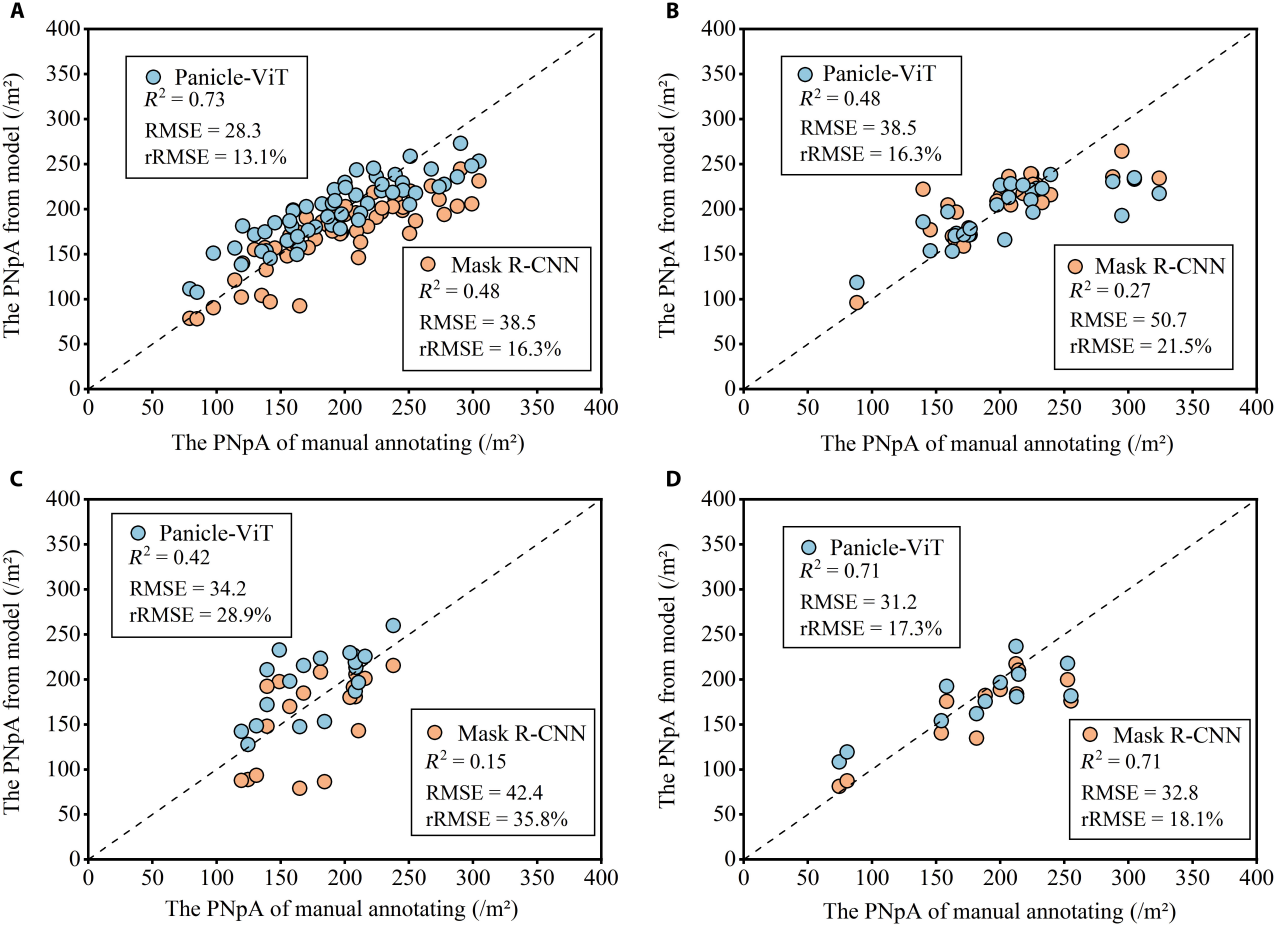
Accuracy of PNpA based on Mask R-CNN (orange points) and Panicle-ViT (blue points). All-stage images (A), the first third stage (B), the second third stage (C), and the final third stage (D). *R*^2^, RMSE, and rRMSE represent the coefficient of determination, root mean square error, and relative RMSE, respectively.

Considering that our ultimate goal is to acquire the phenotypic parameters of rice panicle counts per unit area, evaluating models solely on the basis of their detection accuracy is not comprehensive. We conducted an analysis comparing the PNpA under different backbone networks with manually annotated panicle counts, as shown in Fig. 8. Panicle-ViT outperforms Mask R-CNN across all the datasets, including images from the all-stage images and individual stages. For images spanning the all-stage, the predicted PNpA and the annotated PNpA yielded an *R*^2^ of 0.73, an RMSE of 28.3, and an rRMSE of 13.1%, which is a 26.5% decrease compared with Mask R-CNN. In the first third and second third stages of the FHtM, the rRMSE decreased by 24.2% and 19.3%, respectively. At the final third stage, both models showed consistent *R*^2^ values, with Panicle-ViT’s RMSE at 31.2, which was slightly lower than Mask R-CNN’s RMSE of 32.8, and a 4.4% decrease in the rRMSE.

In the PNpA quantification results across different stages when Mask R-CNN was used, the final third stage of the FHtM exhibited the best performance, with *R*^2^, RMSE, and rRMSE values superior to those of the other 2 stages. When Panicle-ViT was used, the final third stage showed the best performance in terms of *R*^2^ and RMSE, whereas from the perspective of rRMSE, the first third stage surpassed the other 2 stages.

In summary, compared with manually annotated PNpA, using Panicle-ViT as the backbone network for PNpA quantification yields higher accuracy across datasets for all-stage and individual-stage images, surpassing the performance of Mask R-CNN.

### Classification of the 4 panicle types

**Fig. 9.**
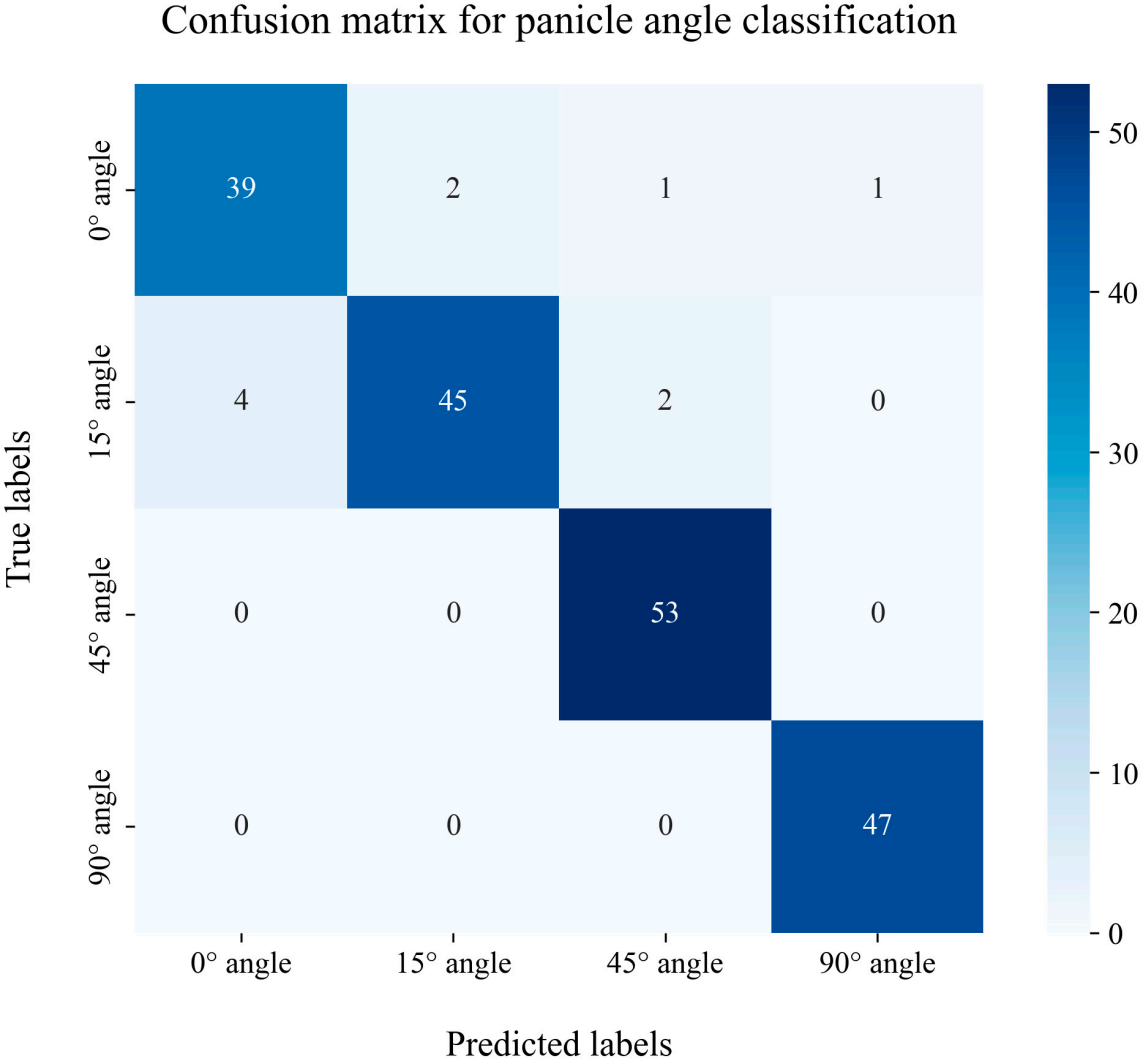
Confusion matrix of the Res2Net50 classifier for the testing dataset.

Figure 9 shows the confusion matrix for classifying 4 types of rice panicle curvatures via the Res2Net50 classifier on the test dataset. The classification model achieved an overall accuracy of 94.85%, with a precision of 94.75%, a recall of 94.73%, and an F1 score of 94.68%. The F1 scores for the 0° angle and 15° angle were relatively low at 90.70% and 91.84%, respectively, whereas the 45° and 90° angles achieved higher F1 scores of 97.25% and 98.95%, respectively, all exceeding 90%. The lower F1 scores for the 0° angle and 15° angle could result from the mutual misclassification between these 2 categories, as indicated in the confusion matrix. This misclassification likely stems from the minimal visual differences between their images, which complicates the classification task, as shown in Fig. 6A and B. In summary, the Res2Net50 classifier effectively categorizes the defined rice panicle curvatures in this study, offering breeders a quantitative way for phenotyping of rice panicle types.

## Discussion

### Trade-off between precision and cost for high-throughput phenotyping of PNpA

**Fig. 10.**
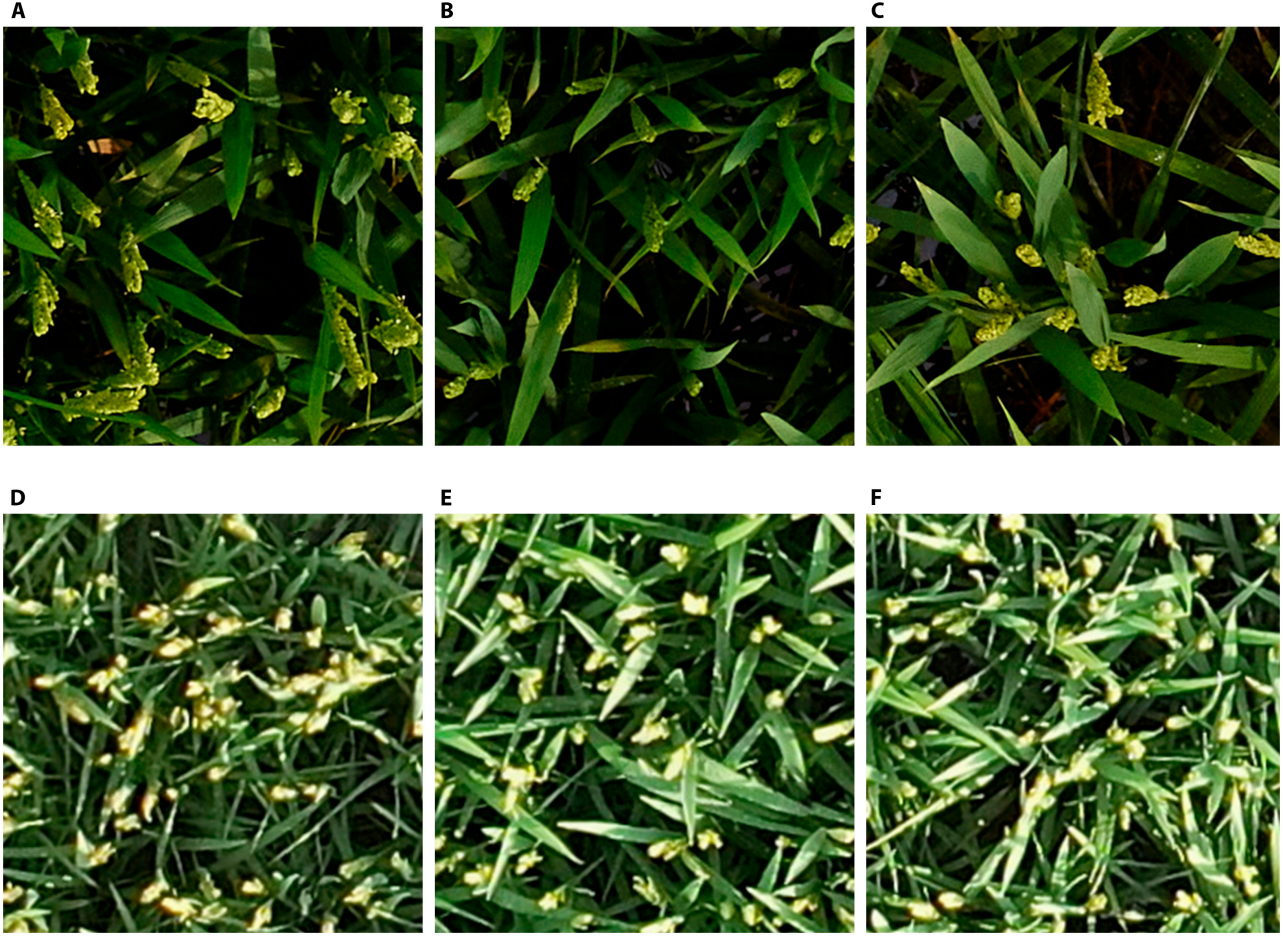
Comparison between ultrahigh-resolution (A to C) and high-resolution images (D to F).

As shown in Table 2, while Panicle-ViT demonstrates improvements in precision metrics compared with Mask R-CNN, the values remain relatively low, primarily because of the resolution of the images used. It is clear that higher spatial resolution images enhance precision. The ultrahigh-resolution dataset was employed for training and testing, and a comparison between the ultrahigh-resolution and high-resolution images is shown in Fig. 10. Using the same training hyperparameters as those for high-resolution images, as detailed in Table 3, the model accuracy metrics for ultrahigh-resolution images are AP_50_ and AR_50_ at 0.824 and 0.850, respectively, and AP_75_ and AR_75_ at 0.586 and 0.663, respectively. This improvement in AP metrics suggests that increasing image resolution enhances model accuracy. This enhancement in detection accuracy stems from the ability of the algorithm to extract more precise features, which facilitates the distinction between the background and the subject more effectively. However, for tasks that do not require perfect object segmentation, such as counting rice panicles, pursuing excessively high-resolution images may not be necessary.

**Table 3.** Evaluation of rice panicle detection accuracy for Panicle-ViT on ultrahigh-resolution datasets

Dataset	Network	AP_50_	AP_75_	AR_50_	AR_75_
Ultrahigh-resolution	Panicle ViT	0.824	0.586	0.850	0.663

The ultrahigh-resolution detection results from Table 3 suggest that using cameras with higher resolution, such as the iXM-100, or capturing images from lower flight altitudes to achieve a higher resolution could improve the model’s predictive performance for PNpA. However, it is crucial to balance operational efficiency, hardware cost, and computational capability, precision, and cost when implementing high-throughput phenotyping of PNpA in practical applications [48,61]. Taking the iXM-100 camera with an 80-mm lens and the P1 camera with a 35-mm lens as examples, iXM-100 has a field of view of 30.4°, whereas P1 has a field of view of 63.5°. Assuming the same altitude and overlap rate for operations, P1 can achieve approximately 5.2 times the operational efficiency of the iXM-100 camera. For the 0.40 ha experimental field in 2022, the operating time with P1 is approximately 10 min, whereas it is 50 min with iXM-100. Additionally, owing to the heavier iXM-100 camera, the endurance of the UAV is reduced to only 15 min of flight time, whereas it is 30 min with the P1. Considering the time required for battery swaps and charging, the operational efficiency of P1 exceeds that of iXM-100 by a factor greater than 5. From a cost perspective, the cost of P1 is approximately ^1^/_10_ that of iXM-100, making it more economically viable for practical applications. In terms of the computational cost, a single raw image from P1 is approximately 20 GB, which is manageable on a consumer-grade PC, whereas a single raw image from iXM-100 is 140 GB, which requires server-level resources for processing.

Although several studies have explored the potential of UAV imaging for rice panicle detection and achieved the desired accuracies, their operational efficiencies are relatively low [22,23,29]. Compared with the reported studies, our campaign efficiency is greater by a factor of 5 to 78, as calculated through the flight planning software WayPoint Master (Weibozhikong, Nanjing, China). The results are shown in Table S2. The proposed approach balances breeding operational efficiency with panicle detection precision and proposes a feasible pipeline from image acquisition to plot segmentation and PNpA quantification, which can be easily adopted by professional and nonprofessional users in practical breeding applications.

### Panicle detection accuracy at different growth stages

Upon analyzing the accuracy of PNpA quantification in Fig. 8B to D, which represent different stages of the FHtM, it is evident that the second third stage showed an inferior performance compared with the other 2 stages. This occurred because, during the second third stage, the rice plants were mostly between the milking and waxing phases, as shown in Fig. S6. At this time, the grains begin to fill, and the panicle gradually becomes plump, displaying a semitransparent, waxy appearance [62]. Since the leaves still need to provide nutrients for the panicle through photosynthesis, they remain green, leading to significant reflection from both the panicles and leaves, which slightly lowers the detection accuracy.

In the first third stage of the FHtM, the rice is mostly between the heading and milking stages, with the panicles just emerging and appearing yellow–green, causing less reflection. However, the leaves reflect more strongly. In the final third stage, the rice is mostly between the waxing and full MSs. The leaves no longer need to perform photosynthesis to provide nutrients and gradually turn yellow–brown, reducing reflectivity, whereas the mature panicles turn golden yellow, reflecting more light. Therefore, during the first third stage and final third stage, the distinction between the panicles and leaves is more pronounced, facilitating the detection of panicles and hence increasing accuracy compared with the second third stage.

### Comparison with manual sampling

As shown in Table S3, the stability results for manual sampling in the plots revealed significant differences, with mean PNpA values from the 2 samples of 306.1 and 272.2 per m^2^ and standard deviations of 61.9 and 42.3 per m^2^, respectively. Moreover, the *P* value from the Wilcoxon test, at 5.1 × 10^−17^, is significantly less than the significance level of 0.05, suggesting that it is statistically improbable for such sampling results to come from the same data distribution. This implies certain issues with the method of manually selecting 6 rice plants to represent the PNpA parameter. Furthermore, the correlation analysis between the 2 samples yielded a correlation value of only 0.47, indicating a moderate positive correlation at best. Furthermore, a regression analysis was conducted to compare the PNpA from the second manual sampling to the PNpA regarded as the true value from the image annotation. The results, as shown in Fig. S1, indicated an *R*^2^ of −2.7, an RMSE of 104.2, and an rRMSE of 52.2%.

Therefore, the method of sampling 6 plants to represent the PNpA of a plot still has some issues, as there is considerable variance between the results of 2 consecutive samplings. This is primarily due to the significant variability among individual rice plants at the early stage, where the number of panicles per plant can differ substantially. Relying on only a few plants for PNpA assessment may not be representative; however, sampling a larger number of plants is labor intensive. UAV imagery offers high-throughput PNpA with superior representativeness for the plot and a more accurate trend for the population.

### Robustness of the PNpA model

**Fig. 11.**
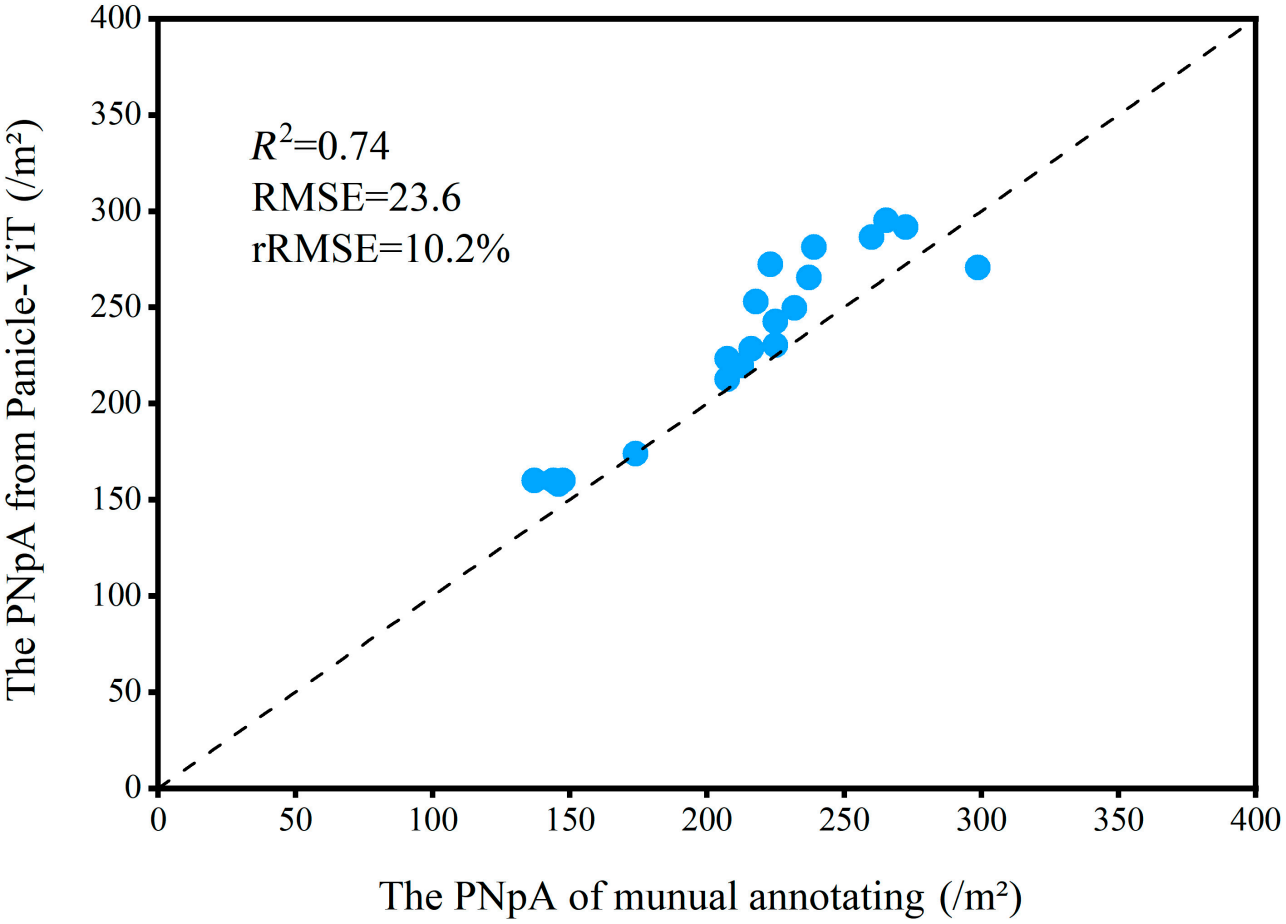
Validation of Panicle-ViT predictions for the PNpA on the basis of the 2023-Val dataset.

Although different breeding materials and planting configurations were used over 2 consecutive years at the 2 experimental sites, the Panicle-ViT model demonstrated robustness in predicting the PNpA. As shown in Fig. 11, the prediction of PNpA on the 2023-Val dataset achieved an *R*^2^ of 0.74, an RMSE of 23.6, and an rRMSE of 10.2%, which closely aligns with the performance obtained from the 2022 dataset. The PNpA model was capable of delivering reliable predictions across varying genetic backgrounds, planting densities, and environmental conditions.

**Fig. 12.**
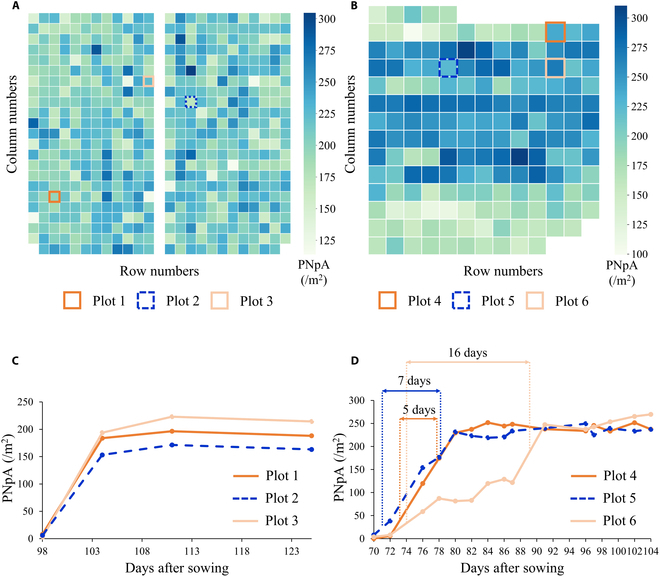
Mapping examples of the predicted PNpA at field 1 in 2022 on day 125 after sowing (A) and at field 2 in 2023 on day 104 after sowing (B), along with the time series analysis for PNpA in 3 plots from field 1 in 2022 (C), and 3 plots from field 2 in 2023, with the left dashed line indicating the day when 10% heading occurred and the right dashed line indicating the day when 80% heading was achieved (D).

Figure 12A and B shows the predicted PNpA across all plots at field 1 in 2022, which was based on UAV images acquired on day 125 after sowing, and at field 2 in 2023, which was acquired on day 104 after sowing. Furthermore, Fig. 12C and D presents the temporal progression of PNpA in 3 randomly selected plots from field 1 in 2022 and field 2 in 2023. The dense data collection intervals in 2023, ranging from 2 to 4 d, enabled us to define the ”duration of heading”—the interval between 10% and 80% heading. This trait is an indicator of synchronous maturation, which is crucial for breeding varieties adaptable to mechanized operations and climate risks. As shown in Fig. 12D, plot 6 has a heading duration of 16 d, which may not be ideal for breeding purposes. Conversely, plot 4, with a heading duration of only 5 d, is considered more favorable for breeding. However, the 2022 data, with its 1- to 2-week intervals, did not allow for accurate calculation of this trait.

Therefore, we recommend employing UAVs to capture RGB images every 2 to 4 d as the rice crop enters the heading phase. Our proposed methods for plot segmentation and PNpA quantification not only determine final PNpA values and identify panicle types but also provide essential insights into the duration of heading.

### Potentials and limitations of the work

Our Plot-Seg and panicle classification methods offer promising applications in UAV image processing for rice breeding. In crop breeding trials, a large amount of breeding material is usually planted across multiple locations, necessitating high-throughput crop phenotyping within short time windows. An end-to-end image processing pipeline for UAV imagery is critical for further enhancing the applicability of the proposed approach to crop breeding practices for both professional and nonprofessional users. Compared with traditional manual plot grid creation, Plot-Seg greatly accelerates UAV image processing through automatic plot segmentation. Furthermore, by integrating the Panicle-ViT algorithm, this study developed a comprehensive pipeline from efficient image acquisition with UAVs through automatic plot segmentation by Plot-Seg to panicle detection. Additionally, the morphological features of rice panicles can be well described by panicle curvature-based classification, which is beneficial for breeding targeted at specific plant structures, such as optimizing panicle architecture to increase light utilization efficiency. This allows breeders to obtain more detailed phenotypic information to support trait selection and breeding strategies [63,64]. However, these methods still face some challenges. Since panicle detection and classification are based on images of the rice canopy, the accuracy of counting and classification could be affected by leaf–panicle overlap and occlusion. Additionally, in one plot, multiple heading stages may occur, especially for the breeding materials, and the proposed approach may not be able to recognize such variation in individual plants within the plot. Future studies on 3-dimensional reconstructions of whole plots could be a potential research direction to further improve the efficiency and accuracy of high-throughput phenotyping of rice panicles.

## Conclusions

This study introduces an automated method for segmenting plots and evaluating PNpAs for use in breeding, which uses UAV imagery and a deep learning instance segmentation algorithm. The results demonstrated that the accuracy of plot segmentation based on the algorithm closely matched that of manual segmentation, with the standard deviation of algorithmic segmentation showing even greater stability than manual segmentation. In terms of PNpA quantification accuracy, Panicle-ViT, which uses a ViT as its backbone network, outperforms the traditional Mask R-CNN across all accuracy metrics for images from both the entire period and each stage of the FHtM, with an improvement in AP_50_ ranging between 3.5% and 20.5%. For panicle count quantification, the Panicle-ViT model predicted the count with an *R*^2^ of 0.73, an RMSE of 28.3, and an rRMSE of 13.1% in the dataset for the entire period, showing a 26.5% reduction compared with Mask R-CNN and highlighting the advantages of Panicle-ViT in dense panicle detection. In the classification task of the 4 panicle types, the Res2Net50 classifier achieved an overall accuracy of 94.8%, indicating excellent classification performance. Additionally, this study explored the accuracy of PNpA quantification across different stages, considering the impact of rice panicle and leaf reflectance at various growth stages on detection. The model’s robustness was validated on the 2023-Val dataset of hybrid rice breeding materials, with accuracy consistent with that of 2022. These findings illustrate that the combination of efficient UAV image collection, automated plot segmentation algorithms, and deep learning instance segmentation models provides a powerful tool for high-throughput evaluation of PNpA parameters in rice breeding, accelerating the pace of smart breeding.

## Data Availability

The data are freely available upon reasonable request.
