# Supplementary material for: Phenotyping of Panicle Number and Shape in Rice Breeding Materials Based on Unmanned Aerial Vehicle Imagery
Source: Plant Phenomics. 2024 Oct 24;6:0265. doi: 10.34133/plantphenomics.0265 (PMC11499587; doi:10.34133/plantphenomics.0265)
Supplement: Supplementary 1 — Figs. S1 to S7 Tables S1 to S3 [file plantphenomics.0265.f1.zip › Supplementary Materials.pdf]

Supplementary Materials

Figures S1 to S7

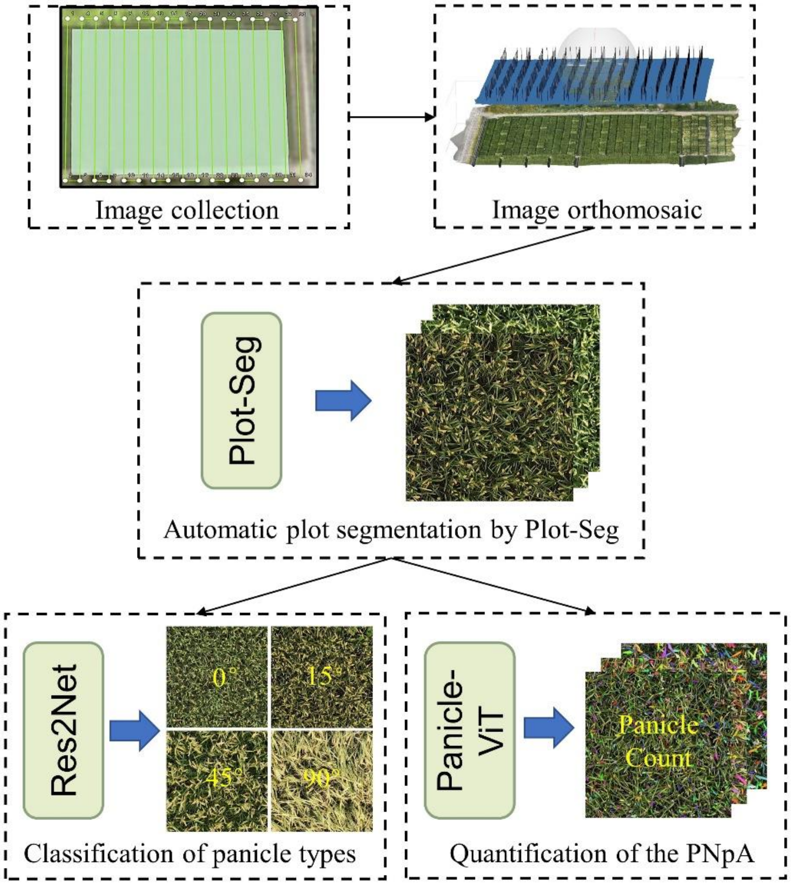

Fig. S1 Workflow for phenotyping panicle number and shape on the basis of UAV images. RGB images captured by UAVs are processed into field orthomosaics. These are then automatically segmented into plot images via Plot-Seg, classified into four panicle types via a Res2Net classifier, and the number of panicles is identified via the Panicle-ViT model.

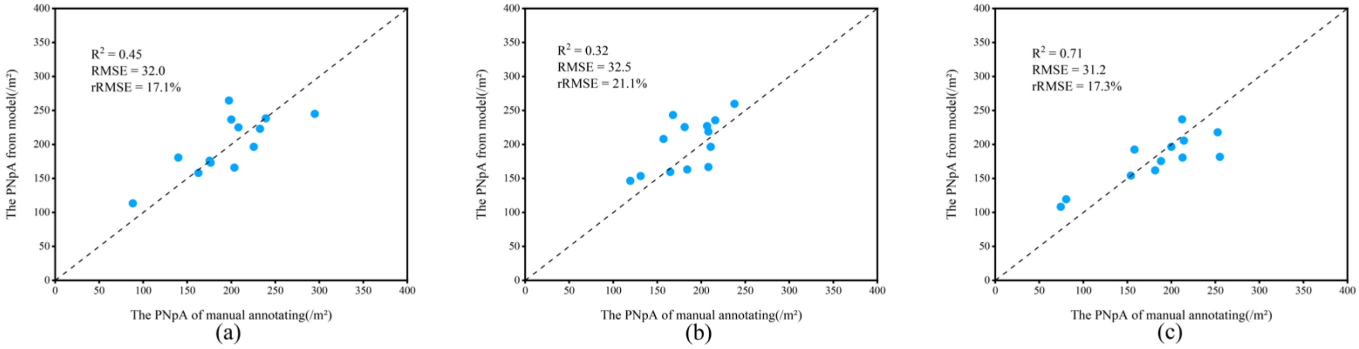

Fig. S2. Accuracy assessment of the number of panicles per unit area (PNpA) via Panicle-ViT across three equal-sized datasets representing different growth stages: the first third stage (a), second third stage (b), and final third stage (c). R^2, RMSE and rRMSE represent the coefficient of determination, root mean square error and relative RMSE, respectively.

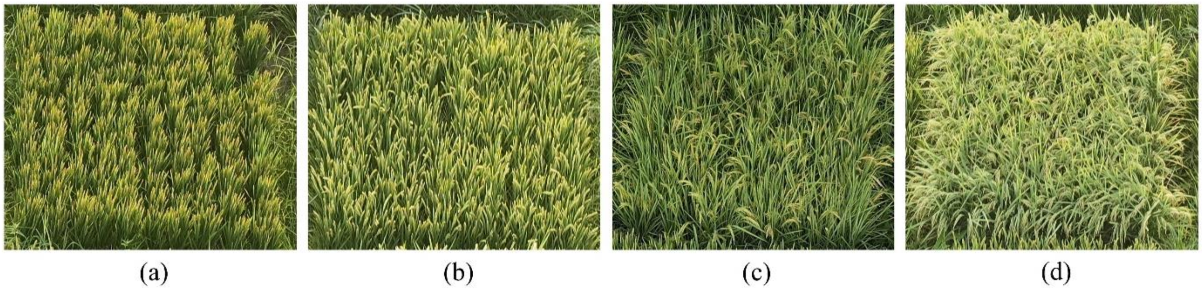

Fig. S3. Oblique photography of panicle types: 0° angle (a), 15° angle (b), 45° angle (c), and 90° angle (d)

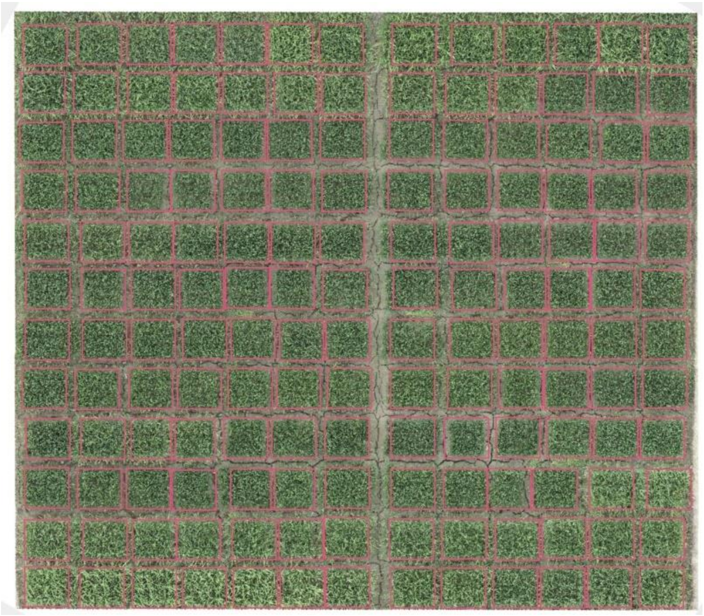

Fig. S4. Plot-Seg segmentation results for Field 2 in 2023, showing the plots aligned vertically and horizontally owing to the algorithm's initial step of orienting the field. This results in a squared appearance, differing from that in Fig. 1b.

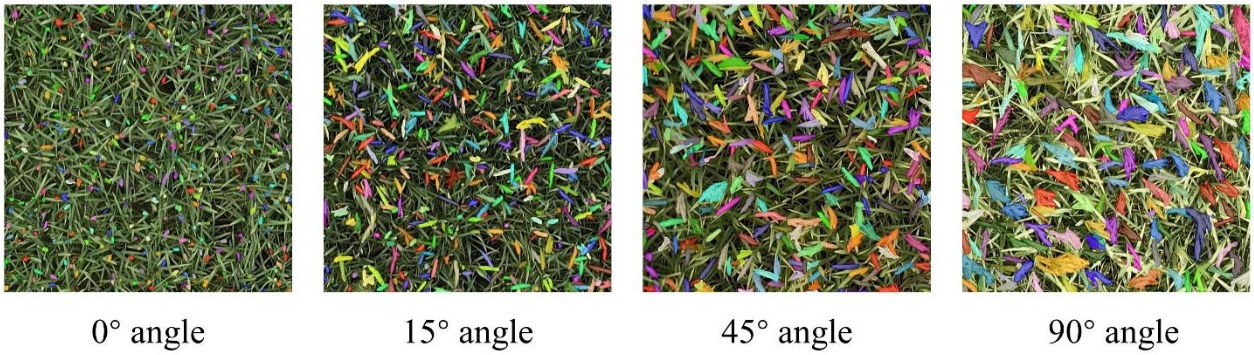

Fig. S5. The visualization of Panicle-ViT for four types of panicles

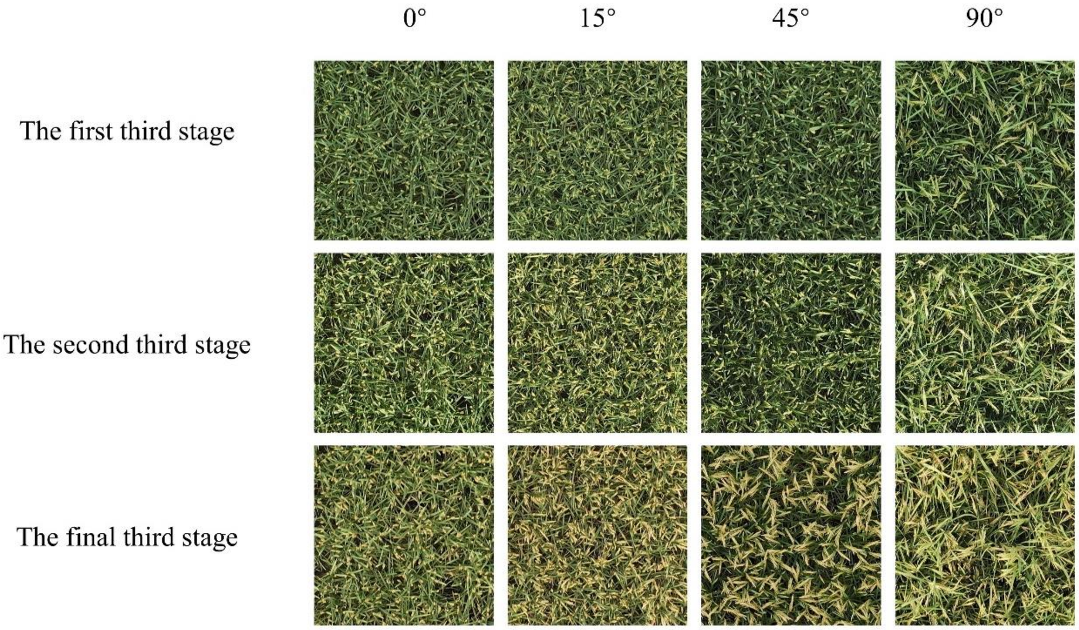

Fig. S6. Four types of angle images at different growth stages.

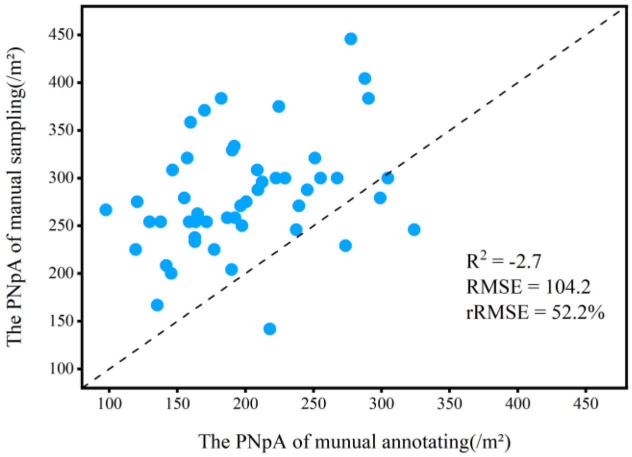

Fig. S7. Relationship between the PNpA of annotated images and manually sampled plots.

Tables S1 to S3

Table S1. Number of annotated images in the rice panicle dataset.

Image dataset	Camera	Experiment site	Total count	Training set	Validation set	Test set
All-stage	P1	Field 1	294	176	59	59
The first third stage			138	86	26	26
The second third stage			96	58	19	19
The final third stage			60	35	13	12
Ultrahigh-resolution	iXM-100		50	30	10	10
2023-Val	P1	Field 2	100	60	20	20

The first third stage, second third stage, and final third stage represent the first third, second third, and final third of the full heading to maturity phase (FHtM), respectively. The ultrahigh-resolution dataset comprises images captured by the iXM-100 camera at Field 1 on Day 98 after sowing in 2022 and is utilized to assess the impact of camera resolution on model accuracy. The 2023-Val dataset includes images obtained from Field 2 on Day 104 after sowing in 2023 and is used for the validation of model robustness.

Table S2. Comparison of operating times for different UAVs and cameras at various altitudes for Field 1

UAV	Camera	Altitude (m)	Lateral and forward overlaps	operating time (minute)
M300	P1	15.0	80% and 70%	10.2
PHANTOM 4 RTK	FC6310R (1)	3.0		102.1
Mavic Pro	/	1.2		784.5
PHANTOM 4 RTK	FC6310R (2)	2.3		158.5
PHANTOM 4 RTK	FC6310R (1)	4.3		48.5

Owing to our inability to obtain a variety of UAVs and cameras, we employed a flight planning software named WayPoint Master (Weibozhikong, Nanjing, China) to simulate the operational times of different UAVs equipped with various cameras at different altitudes. FC6310R (1) and FC6310R (2) each denote two different specifications of photo modes. In the planning process, the interval between camera shots was consistently maintained.

Table S3. Stability analysis of the PNpA by two rounds of manual sampling in plots

Number of plots	Mean	SD	Wilcoxon test	*P*-value	Correlation
222	306.1	61.9	3954.0	5.1×10^{-17}	0.47
222	272.2	42.3			
