# Supplementary figures and images for: Phenotyping of Panicle Number and Shape in Rice Breeding Materials Based on Unmanned Aerial Vehicle Imagery
Source: Plant Phenomics. 2024 Oct 24;6:0265. doi: 10.34133/plantphenomics.0265 (PMC11499587; doi:10.34133/plantphenomics.0265)

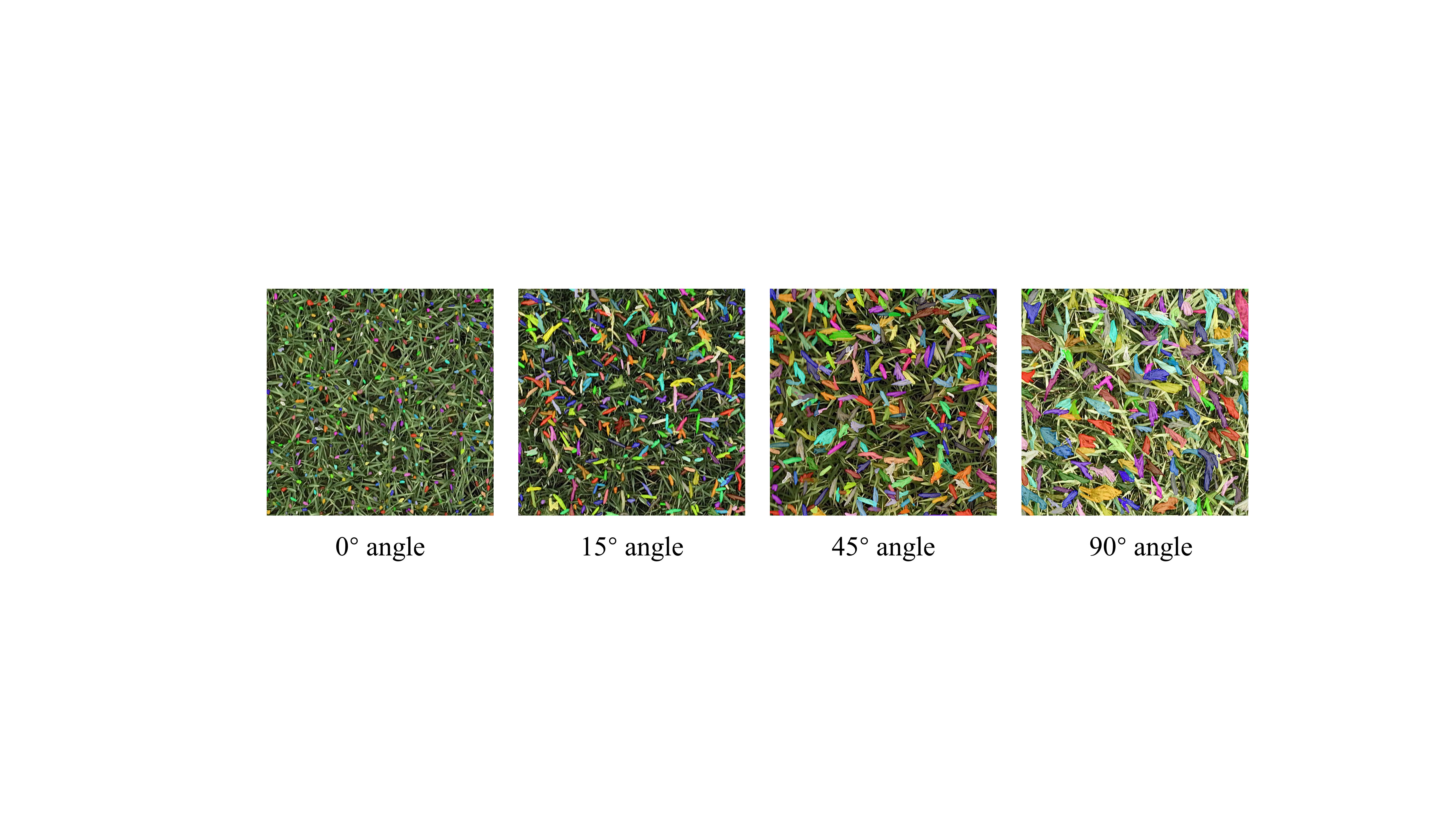

Supplement: Supplementary 1 — Figs. S1 to S7 Tables S1 to S3 [file plantphenomics.0265.f1.zip › Fig. S5.jpg]

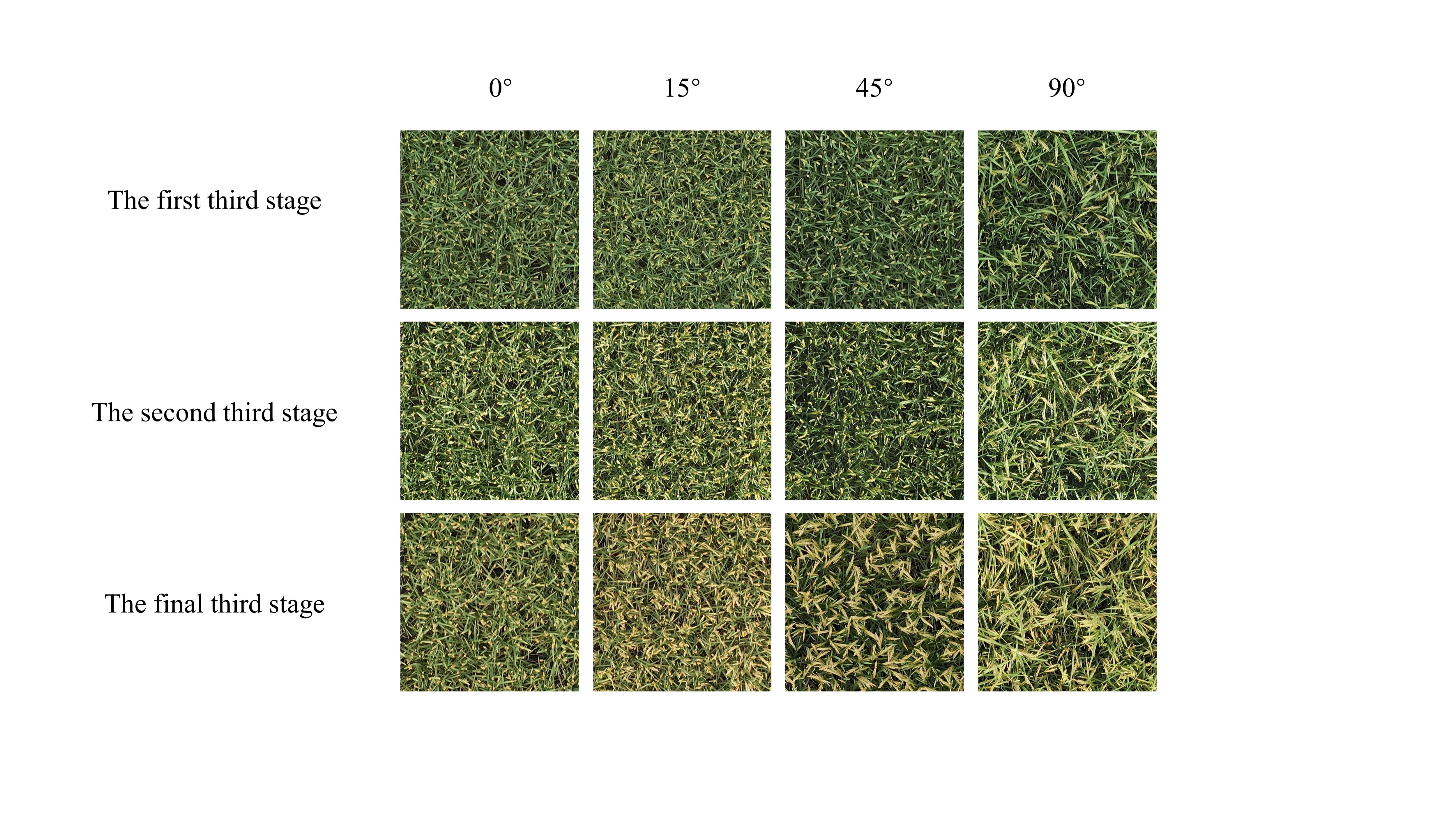

Supplement: Supplementary 1 — Figs. S1 to S7 Tables S1 to S3 [file plantphenomics.0265.f1.zip › Fig. S6.jpg]

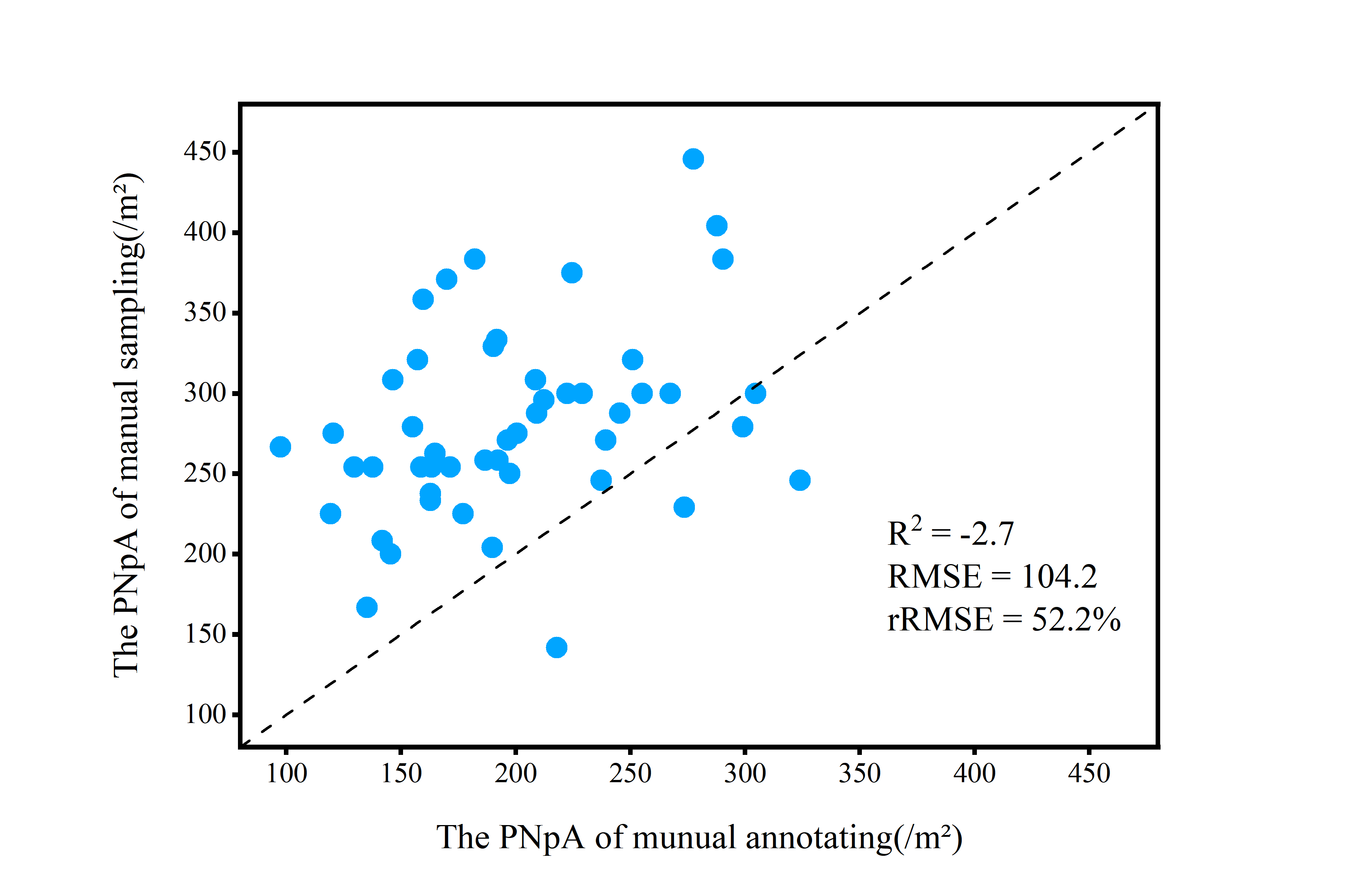

Supplement: Supplementary 1 — Figs. S1 to S7 Tables S1 to S3 [file plantphenomics.0265.f1.zip › Fig. S7.png]
